# Diagnostic accuracy of ultrasound superb microvascular imaging for focal liver lesions: A protocol for systematic review and meta-analysis

**DOI:** 10.1097/MD.0000000000024411

**Published:** 2021-01-22

**Authors:** Ping Sui, Xiaoyan Wang, Lipeng Sun, Hui Wang

**Affiliations:** Ultrasound Department of the First Affiliated Hospital of Dalian Medical University, Liaoning Province, China.

**Keywords:** focal liver lessions, meta-analysis, superb microvascular imaging

## Abstract

**Background:**

Superb microvascular imaging (SMI) is a new ultrasound vascular imaging technology, which uses a new Doppler algorithm, it has the characteristics of high sensitivity and high resolution to detect low velocity blood flow; it is easier to detect microvessels with low-velocity flow compared with color Doppler flow imaging in theory; and it can image the microvessels of the lesion without angiography.^[1]^ Previous studies showed that SMI can detect tumor neovascularization to differentiate benign from malignant focal liver lessions (FLLs). However, the results of these studies have been contradictory with low sample sizes. This meta-analysis tested the hypothesis that SMI is accurate in distinguishing benign and malignant FLLs.

**Methods:**

We will search PubMed, Web of Science, Cochrane Library, and Chinese biomedical databases from their inceptions to the November 30, 2020, without language restrictions. Two authors will independently carry out searching literature records, scanning titles and abstracts, full texts, collecting data, and assessing risk of bias. Review Manager 5.2 and Stata14.0 software will be used for data analysis.

**Results:**

This systematic review will determine the accuracy of SMI in the differential diagnosis between benign and malignant FLLs.

**Conclusion:**

Its findings will provide helpful evidence for the accuracy of SMI in the differential diagnosis between benign and malignant FLLs.

**Systematic review registration:**

INPLASY2020120081.

## 1 Introduction

HCC is the second most common cause of mortality from cancer,^[2,3]^ although the detection rate of FLLs is clearly increasing because of the widespread application of imaging techniques, especially ultrasound examinations,^[4]^ the current diagnostic challenge is how to distinguish between malignant and benign FLLs effectively.^[5,6]^ Contrast-enhanced ultrasound (CEUS) has been gradually recognized as a comparable imaging technique to contrast-enhanced computed tomography (CECT) and contrast-enhanced magnetic resonance imaging (CEMRI) in the diagnosis of FLLs,^[7,8]^ but they are expensive and time-consuming, limiting their widespread use.

To address these challenges, it is essential to establish a diagnostic method that is inexpensive, easy to operate, and has high diagnostic accuracy. SMI is a novel Doppler technique developed by Toshiba Medical System (Tokyo, Japan),^[9,10]^ which was designed to simulate CEUS by using advanced clutter elimination to obtain only vascular flow signals without using any contrast agents.^[11]^ Similar to color Doppler and power Doppler imaging, SMI can provide a real-time examination of vascularity in FLLs, but it has the additional advantages of detecting slower blood flow and revealing micro-vessels.^[4]^ This work will systematically evaluate the technical performance and accuracy of SMI for differential diagnosis of benign and malignant FLLs.

## 2 Materials and methods

This study was conducted in accordance with the PRISMA (Preferred Reporting Items for Systematic Reviews and Meta-Analyses) guidelines and the protocol was registered in the INPLASY (INPLASY2020120081).

### 2.1 Eligibility criteria

#### 2.1.1 Type of study

This study will only include high quality clinical cohort or case control studies.

#### 2.1.2 Type of patients

The patients should be those who had undergone FLLs.

#### 2.1.3 Intervention and comparison

This study compare SMI with pathology for diagnosing FLLs.

#### 2.1.4 Type of outcomes

The primary outcomes include sensitivity, specifificity, positive and negative likelihood ratio, diagnostic odds ratio, and the area under the curve of the summary receiver operating characteristic.

### 2.2 Search methods

PubMed, Web of Science, Cochrane Library, and Chinese biomedical databases will be searched from their inceptions to the November 30, 2020, without language restrictions. The search strategy for PubMed is shown in Table 1. Other online databases will be used in the same strategy.

**Table 1:** Search strategy sample of PubMed.

Number	Search terms
1	focal liver lessions or hepatic tumors or hepatocellular carcinoma
2	superb microvascular imaging or superb micro-vascular imaging or SMI
3	Pathology
4	and 1–3

### 2.3 Data extraction and quality assessment

Two authors will independently select the trials according to the inclusion criteria, and import into Endnote X9. Then remove duplicated or ineligible studies. Screen the titles, abstracts, and full texts of all literature to identify eligible studies. All essential data will be extracted using previously created data collection sheet by 2 independent authors. Discrepancies in data collection between 2 authors will be settled down through discussion with the help of another author. The following data will be extracted from each included research: the first authors surname, publication year, language of publication, study design, sample size, number of lesions, source of the subjects, instrument, “gold standard,” and diagnostic accuracy. The true positives, true negatives, false positives, and false negatives in the fourfold (2 × 2) tables were also collected. Methodological quality was independently assessed by 2 researchers based on the quality assessment of studies of diagnostic accuracy studies (QUADAS) tool. The QUADAS criteria included 14 assessment items. Each of these items was scored as “yes” (2), “no” (0), or “unclear” (1). The QUADAS score ranged from 0 to 28, and a score ≥22 indicated good quality. Any disagreements between 2 investigators will be solved through discussion or consultation by a 3rd investigator.

### 2.4 Statistical analysis

The STATA version 14.0 (Stata Corp, College Station, TX, USA) and Meta-Disc version 1.4 (Universidad Complutense, Madrid, Spain) softwares were used for meta-analysis. We calculated the pooled summary statistics for sensitivity, specifificity, positive and negative likelihood ratio, and diagnostic odds ratio with their 95% confidence intervals. The summary receiver operating characteristic curve and corresponding area under the curve were obtained. The threshold effect was assessed using Spearman correlation coefficients. The Cochrans Q-statistic and I test were used to evaluate potential heterogeneity between studies. If significant heterogeneity was detected (Q test *P* < .05 or *I* test >50%), a random effects model or fixed effects model was used. We also performed sub group and meta-regression analyses to investigate potential sources of heterogeneity. To evaluate the influence of single studies on the overall estimate, a sensitivity analysis was performed. We conducted Beggs funnel plots and Eggers linear regression tests to investigate publication bias.

### 2.5 Ethics and dissemination

We will not obtain ethic documents because this study will be conducted based on the data of published literature. We expect to publish this study on a peer-reviewed journal.

## 3 Discussion

HCC is one of the most common cancers worldwide,^[12]^ and its accurate differentiation is important for clinical decision-making. The ultrasound features of hepatic malignant lessions include an unclear margin, irregular shape, abundant blood supply, and satellite nodules. These findings increase the likelihood that a focal liver lession is malignant, but they can not independently diagnose malignant lessions. As a novel ultrasonic technique, SMI can not only provide a real-time examination of vascularity in FLLs, but also detect slower blood flow and reveal micro-vessels, which is one of the most important elements of the tumor microenvironment,^[13]^ plays an important role in the development and progression of lesions, and is essential for their differential diagnosis. The blood flow distribution patterns of benign and malignant FLLs are different,^[4,14]^ so the SMI technical can be used to assess the microvascular morphology of FLLs to provide additional diagnostic information. This work will systematically evaluate the technical performance and accuracy of SMI for differential diagnosis of benign and malignant FLLs.

## Author contributions

**Conceptualization:** Lipeng Sun, Hui Wang.

**Data curation:** Ping Sui, Xiaoyan Wang.

**Methodology:** Ping Sui, Xiaoyan Wang.

**Writing – original draft:** Ping Sui.

**Writing – review & editing:** Ping Sui, Hui Wang.
